# Comment on Jaworska, J. et al. Consensus on the Application of Lung Ultrasound in Pneumonia and Bronchiolitis in Children. *Diagnostics* **2020**, *10*, 935

**DOI:** 10.3390/diagnostics11010055

**Published:** 2021-01-04

**Authors:** Raffaella Nenna, Elio Iovine, Marco Laudisa, Silvia Bloise, Domenico Paolo La Regina, Fabio Midulla

**Affiliations:** Department of Maternal Infantile and Urological Sciences, Sapienza University of Rome, Rome 00161, Italy; elio.iovine@gmail.com (E.I.); marco.laudisa1994@gmail.com (M.L.); silvia.bloise1989@gmail.com (S.B.); laregina.domenico@gmail.com (D.P.L.R.); midulla@uniroma1.it (F.M.)

To the editor:

We have read with great interest the article by Jaworska et al. [1] concerning the role of Lung Ultrasound (LUS) in pneumonia and bronchiolitis in children.

These diseases are very common, and especially in the pediatric population it is necessary to find a bedside diagnostic method able to reach and eventually overcome the effectiveness of chest-X-Ray (CXR) in the diagnosis and follow-up of these diseases, so we reckon that the topic is undoubtedly of great interest. The statements remarked in the consensus, together with more recent evidence [2,3], will certainly represent a solid basis for the introduction of LUS in future guidelines for the diagnosis and management of respiratory tract infections in children. LUS has outstanding advantages: it enables bedside assessment, prevents exposure to radiation, reduces the length of patient stay in the emergency room, can be repeated during follow-up at low cost and is easily applied also in low-resource settings. However, as an aerated lung blocks the transmission of ultrasound, LUS cannot detect lesions that are deep within the lung [4].

Hereby, we would like to report that there are additional factors that should be taken into account in the recommendation for LUS in pediatric respiratory infections. First of all, the timing of performing LUS has a primary importance in order to evaluate the effectiveness of the method, and it would be desirable to introduce the optimal timing as an indication in the application of LUS.

Actually, timing in performing LUS was controlled in our recent studies [2,3], in which LUS was performed in children with bronchiolitis or pneumonia, only within the first 24 h from admission in the Emergency department. 

It is also interesting to note that, we not only confirmed the positive correlation between the severity of bronchiolitis and LUS findings, but also identified a positive correlation between the severity of LUS findings and the length of hospitalization [2]. This further confirms the statements of the consensus about the role of LUS in the diagnosis and management of bronchiolitis, strengthening the evidence of a correlation between the severity of the disease and the involvement of the lung in LUS.

We also confirmed with a recent study [3] the role of LUS in the diagnosis of pneumonia, highlighting the role of LUS especially in finding complications of the disease. In this study, the LUS was able to detect not only pleural effusion but also, in one patient, an intra parenchymal small abscess cavity and at an earlier stage than CXR. The presence of the abscess cavity was later confirmed with a computed tomography scan of the lung, testifying the superiority of LUS compared with CXR in detecting this kind of complication of the disease.

We acknowledge the important work of Jaworska et al. and we hope that, according to the growing evidence, LUS will be soon introduced in new guidelines for the diagnosis and management of pneumonia and bronchiolitis in children.

## Data Availability

Not applicable.
